# A chaotic spiking backpropagation approach to brain learning

**DOI:** 10.1093/nsr/nwae070

**Published:** 2024-03-04

**Authors:** Guanrong Chen

**Affiliations:** Department of Electrical Engineering, City University of Hong Kong, China

## Abstract

A highlight of the chaotic spiking backpropagation (CSBP) method, which is a powerful tool for directly training spiking neural networks and helps to understand the learning mechanisms of human brain.

Spiking neural networks (SNNs) [1] are known for their superior energy efficiency, achieved through spiking signal transmission that mirrors biological nervous systems, thus solving the unsustainable energy-consumption problem of current artificial neural networks and frameworks. However, effectively training SNNs poses a significant technical challenge. While surrogate gradient-based methods provide a viable solution, the trained SNNs often get stuck in local minima due to their reliance on the inherent gradient dynamics.

Skarda and Freeman's experiments in 1987 showed that chaotic dynamics [2] of brain neurons are indispensable when it comes to rabbits acquiring new olfactory patterns [3]. Then, Matsumoto *et al.* found that the nervous system of a squid perceives external information based on chaotic dynamics [4]. These studies suggest that, with millions of years of evolution, animal brains exploit the chaotic dynamics of brain neurons for information processing.

Drawing inspiration from the chaotic dynamics observed from brain learning, Chen's group introduce the chaotic spiking backpropagation (CSBP) method [5]. This method incorporates a loss function to emulate brain-like chaotic dynamics, leveraging its ergodic and pseudo-random nature to enhance the effectiveness and robustness of SNN-based learning. Such a learning process starts from a global chaotic itineracy with searching fluctuations of the states, then undergoes various bifurcations with rich canalization dynamics, and eventually converges locally to a fixed point corresponding to the learning result.

The paper [5] not only presents a powerful tool for directly training SNNs but also provides fresh insights into the learning mechanisms of the human brain, characterized by the CSBP with the following key attributes:

- CSBP inherently integrates chaotic dynamics inspired by animal brains.
- CSBP theoretically ensures global search capability and convergence.
- CSBP surpasses current state-of-the-art methods, excelling not only with neuromorphic data sets like DVS-CIFAR10 and DVS-Gesture but also with large-scale static data sets such as ImageNet and CIFAR100.
- CSBP can function as an add-on or plug-in module to any existing method by simply introducing one term, thereby enhancing performances without altering the parameters of the original method.
- CSBP demonstrates robustness with respect to both initial conditions and hyperparameters, mitigating the persistent issue of trapping into local minima encountered by existing methods.
